# Addressing the autism mental health crisis: the potential of phenomenology in neurodiversity-affirming clinical practices

**DOI:** 10.3389/fpsyg.2023.1225152

**Published:** 2023-09-04

**Authors:** Themistoklis Pantazakos, Gert-Jan Vanaken

**Affiliations:** ^1^Department of Science and Technology Studies, University College London, London, United Kingdom; ^2^The American College of Greece, Athens, Greece; ^3^Parenting and Special Education Research Unit, Faculty of Psychology and Educational Sciences, KU Leuven, Leuven, Belgium; ^4^Leuven Autism Research, KU Leuven, Leuven, Belgium; ^5^Department of Philosophy, Centre for Ethics, University of Antwerp, Antwerp, Belgium

**Keywords:** autism, neurodiversity, phenomenology, psychotherapy, mental health

## Abstract

The neurodiversity movement has introduced a new era for autism research. Yet, the neurodiversity paradigm and the autism clinic remain largely unconnected. With the present work, we aim to contribute to filling this lacuna by putting forward phenomenology as a foundation for developing neurodiversity-affirming clinical interventions for autism. In the first part of this paper, we highlight that autistic people face a severe mental health crisis. We argue that approaches focused on reducing autistic ‘symptoms’ are unlikely to solve the problem, as autistic mental health is positively correlated with autism acceptance and perceived quality of support provided, not necessarily with lack of ‘symptomatologic severity’. Therefore, the development and dissemination of neurodiversity-affirming clinical interventions is key for addressing the autism mental health crisis. However, therapists and researchers exploring such neurodiversity-affirming practices are faced with two significant challenges. First, they lack concrete methodological principles regarding the incorporation of neurodiversity into clinical work. Second, they need to find ways to acknowledge rightful calls to respect the ‘autistic self’ within the clinic, while also challenging certain beliefs and behaviors of autistic clients in a manner that is *sine qua non* for therapy, irrespective of neurotype. In the second part of the paper, we introduce phenomenological psychology as a potential resource for engaging with these challenges in neurodiversity-affirming approaches to psychotherapy. In this vein, we put forward specific directions for adapting cognitive behavioral and interpersonal psychotherapy for autism.

## 1. Introduction

Born during the peak of the U.S. civil rights movement in the 1960s, the disability rights movement surfaced with a unified front for combating the abuse and neglect of people with disabilities, and for overcoming barriers that stand between them and a good human life (Sabatello, 2014). The neurodiversity movement is a recent iteration of the disability rights movement that is chiefly associated with autism, promoting a more affirming view of cognitive diversity and emphasizing multifarious social exclusion (e.g., attitudinal, environmental, institutional) as the principal cause of the adversities neurodivergent people experience (Ne’eman and Pellicano, 2022; Arnaud and Gagné-Julien, 2023). Despite rapidly gaining traction within both academia and popular discourse, the neurodiversity paradigm remains largely unconnected with clinical work. In the present paper, we address this lacuna by first arguing that there is an urgent need for neurodiversity-affirming clinical interventions for autism. Second, we develop a proposal of how phenomenological psychology can ground the adaptation of related therapeutic methods to autistic clients.

Following this introductory section, in Section 2, we provide a brief overview of autism as conceptualized by the medical model, as well as of the neurodiversity movement’s historical roots, central positions, and principles for the autism clinic. In Section 3, we highlight that autistic people face a severe mental health crisis, as they are much more likely to suffer from most major mental disorders and alarmingly more likely to commit suicide compared to the general population (Hirvikoski et al., 2016; Lai et al., 2019). We draw on clinical evidence to claim that autism in itself is unlikely to be the culprit for this, as autistic mental health is correlated with autism acceptance and perceived quality of support given, not primarily with lack of symptom severity. In contrast, clinical interventions that target autistic symptomatology are still among the most frequently delivered ones, all while such symptomatic interventions are extremely understudied regarding their capacity to improve autistic mental health (Harvey et al., 2009). Moreover, autistic people’s experiences within the mental health system are often disheartening. Sometimes these encounters are even associated with counter-productive results as stigma and misunderstanding of autistic clients are still prevalent among practitioners (Crane et al., 2019). Consequently, there is a strong case to be made for mental health support to autistic people that is neurodiversity-affirming in nature. Moreover, the successful implementation of such neurodiversity-affirming care constitutes an urgent social responsibility.

In section 4, we delve into neurodiversity-affirming principles for good clinical practice, departing from two observations. First, current neurodiversity frameworks consist more of ethical guidelines than of concrete methodological principles. To build the bridge to systematic and effective neurodiversity-affirming interventions, these methodological gaps must be filled in. Second, the purpose of said guidelines is chiefly to safeguard human rights within clinical contexts via ascertaining consent, validating autistic self-narratives, and respecting autistic behavioral and cognitive modes within the clinic. While understandable in a landscape awash with abuse-related controversies, this orientation clashes with something fundamental about treatment, especially regarding psychotherapy. Successful mental health interventions, that is, most often necessitate both challenging the client’s self-narrative and cognitive tropes, and/or encouraging them to progressively engage in behaviors that run contrary to their habitual modus operandi (Beutler et al., 2002). Thus, we suggest, a delicate balance must be found between procuring results meaningful in the terms of autistic clients and reserving enough space for fruitfully challenging them. We put forward a clinical-phenomenological approach that seeks to serve this double purpose while solidifying inclusivity buzzwords into concrete clinical implementations. Our hypothesis is that clinical schemes resulting from this research program will succeed in improving autistic subjective wellbeing and providing critical aid in ameliorating the autistic mental health crisis.

To ground our proposal to clinical practice, in section 5 we provide indicative yet tangible examples of how the phenomenological approach may inform two of the most common and effective strands in psychotherapy today, namely cognitive-behavioral therapy and interpersonal psychotherapy. We also comment on our proposal’s limitations, including its applicability to alexithymic and intellectual disabled autistics, and we provide a few overarching comments. In section 6, we offer the paper’s key conclusions.

## 2. Neurodiversity-affirming clinical principles

The Center for Disease Control and Prevention reported that in 1 in 36 children in the U.S. was diagnosed with autism in 2020, with males being about four times as many as females (Center for Disease Control and Prevention, 2023). In clinical practice autism is chiefly operationalized as a neurodevelopmental disorder characterized by issues with communication and sociability, restricted and repetitive behaviors, and/or multimodal sensory sensitivities (American Psychiatric Association, 2013). Autism is understood to be a spectrum condition, ergo the term autism spectrum disorder (ASD). In the disorder framing of the medical model of autism, autism is something that one has, much as one may have arthritis or a brain tumor, hence the oft encountered term ‘person with autism’. By the medical approach, it is neurobiological and psychological dysfunctions within the individual that give rise to autism, and thus to behavioral impairments (Frith, 2003; Baron-Cohen, 2008). An influential conceptualization of autism posits an empathy deficit as a core feature purportedly stemming from an absent or faulty theory of mind, or the ability to correctly ascribe mental states to others (Baron-Cohen, 2008). This view, however, has become increasingly untenable under the scrutiny of research demonstrating both that autistic people do exhibit empathetic understanding of one another and that neurotypical people also face problems with ascribing mental states to autistic people, which constitutes the ‘double empathy problem’ (Milton, 2012). An increasing number of recent accounts signify a departure from cashing out autistic differences in terms of such cognitive divergence, while approaches that prioritize the perceptual differences, well-evidenced to occur in autism, are gaining ground (Markram and Markram, 2010; van Es and Bervoets, 2022).

The concept of neurodiversity was conceived in the mid-1990s within online communities that facilitated connection and sharing of ideas between autistic people around the globe (Singer, 1999; Dekker, 2020). The neurodiversity movement grew to prominence in parallel with significant developments in online communication (Kras, 2009) and is today thought to have succeeded in ushering in a paradigm shift in autism research (Pellicano et al., 2018; Pellicano and Den Houting, 2022). Besides autism, the neurodiversity movement encompasses social groups with other neurocognitive, developmental, and psychological disabilities like attention deficit disorder and dyslexia (Dekker, 2020). While the neurodiversity paradigm’s theses are still a matter of debate even among its theorists and activists, the movement is minimally defined by its opposition to the medical model of disability (Dwyer, 2022). Neurodiversity advocates promote non-pathologization of mental disabilities, acceptance of diversity in how human minds engage with and experience the world, and an understanding of neurominorities as morally equal but marginalized groups justifying an emancipatory struggle (Armstrong, 2015; Vanaken, 2023). In this vein, the neurodiversity movement most often espouses the terms ‘disability’ instead of ‘disorder’ and, in the case of autism, ‘autistic person’ instead of ‘person with autism’ to reflect autism as an individual core feature inseparable from the self (Taboas et al., 2023). Neurodivergent impairment and distress are thus located not in individual dysfunctions, but in an illness of fit between individual and society, often understood in terms of societal barriers that marginalize neurodivergent people. By extension, the neurodiversity movement campaigns against the social exclusion of neurodivergent people, putting forward measures such as self-advocacy, institutional inclusion, neurodiversity related education of society, and participatory research (Nicolaidis, 2012; Leadbitter et al., 2021). Chapman (2016) has claimed that autism is, beyond a medical diagnosis, also a political identity, not necessarily in the sense of self-determination but by way of structural exclusion based on personal characteristics. This, according to Chapman, renders autistic individuals akin to gender, sexual, ethnic, and other minorities who are systemically and systematically marginalized.

Neurodiversity scholars often take connection with clinical practice to be one of the movement’s most urgent priorities (Chapman and Botha, 2022). The chief purpose of this paper is to contribute to this endeavor by mobilizing methods from phenomenological psychology. First, however, it should be noted that the neurodiversity paradigm, despite the general traction it currently enjoys, is still not being proportionately invited to the field of clinical research. A close look at the figures of autism funding allocation reveals a protracted under-prioritization of research into the needs of autistic people and services tailored to them. The 2018 portfolio analysis by the Office of Autism Research Coordination on behalf of the Interagency Autism Coordination Committee (IAAC), the primary advisory body to the U.S. government on issues regarding autism, found that just 6% of both private and public funds went toward researching the quality of services for autistic people, while a mere 2% were utilized in researching the needs of autistic adults. In contrast, the largest piece of the funding pie (39%) went toward researching the biological mechanisms underlying autism (Interagency Autism Coordinating Committee, 2018), which is research principally exploitable by interventions seeking to overturn autism. In a statement relating to a previous report by the same agency and showing similar numbers, Ne’eman, then president of the Autistic Self Advocacy Network, declared that such figures “show a shocking lack of interest in aligning scientific investment in autism research to the priorities of the most important stakeholders: autistic people ourselves” (Autism Self Advocacy Network, 2016). Notably, the IAAC itself has reacted to this state of affairs, recommending a paradigm shift in research orientation with the aim of improving services across the lifespan and addressing the daily needs of autistic people (Interagency Autism Coordinating Committee, 2017). Recently surfacing neurodiversity-affirming therapist collectives and educational tools (Neurodivergent Therapists, 2023; Salvensen Mindroom Research Centre, 2023; Therapist Neurodiversity Collective, 2023) may be regarded as a move in this direction. Despite such bottom-up, pioneering attempts, we may, to repeat, not speak of systematic intervention methods that are concretely neurodiversity-affirming, but at most of recommendations that different lines of neurodiversity research have put forward. In a recent account, Chapman and Botha (2022) group these recommendations under three principles: incorporating standpoint epistemology; resisting normalization; and implementing environmental interventions.

Incorporating standpoint epistemology is founded on undoing the exclusion of autistic people from knowledge production and treatment orientation, and reintroducing them in these processes as equal partners, if not figures of ultimate authority (Chapman and Botha, 2022). Prejudices that have sustained a state of exclusion in the past, such as the purported cognitive deficits and consequent lack of epistemic authority on behalf autistic people, now face mounting counterevidence (Gillespie-Lynch et al., 2017). Beyond shared decision making, standpoint epistemology proffers that clinical practitioners attempt to arrive at a critical consciousness about the methods and goals of the clinic, informed by the social position and own experiences of clients themselves. Chapman and Botha (2022) thus invite epistemic humility on behalf of neurotypical researchers and clinicians, a stance that appears to be applauded by autistic individuals (Hume, 2022).

Resisting normalization, revolves mainly around opposition to interventions that aim to foster ‘normal’ behavior in autistic people. Social skills training can be one such example. Yet the most commonly chosen target for critique here is applied behavior analysis (ABA). ABA was invented by Lovaas and colleagues in the 1960s, with Lovaas (1974, 76) himself viewing autistic children as “not persons in the psychological sense.” Thus, ABA’s initial aim was to tweak such children’s behaviors to fit societal norms, thereby reinstating their personhood. ABA has carved a well-recorded historical trajectory of abuse and violation of human rights, employing tools which have since become widely condemned as illegitimate, such as shouting, slapping, and delivering electric shocks to meet set goals (Bowman and Baker, 2014). Today, the many iterations of ABA have distanced themselves from such techniques and, at least declaratively, largely from aversive methods as well (Schuck et al., 2022), though not without exception (Brown, 2014). Nonetheless, ABA is still self-described as “using scientific principles and procedures discovered through basic and applied research to improve socially significant behavior to a meaningful degree” (Association of Professional Behavior Analysts, 2023). Wilkenfield and McCarthy (2020, 37) characterize the method as one “in which the autistic child is rewarded for engaging in activities that make him more normal.” Expectedly, ABA has consistently found itself in the crosshairs of neurodiversity proponents, who blame the method for attempting to eradicate the autistic self and force autistic people in a neurotypical mold. They contend that ABA attempts to ‘re-program’ autistic individuals, forcing them to leave perfectly harmless behaviors of, e.g., self-regulation behind (Milton and Sims, 2016; Kapp et al., 2019), as well as to take on new behaviors that do not resonate from a first-person point of view, thus failing to improve their wellbeing. For example, many autistic adults often claim that eradication of stimming (self-stimulation) leaves them deprived of an invaluable tool for self-regulation and a part of their identity (Rudy, 2018). Similarly, they find the forced acquisition of typical social behaviors such as eye contact to be thoroughly distressing, even after integration (Trevisan et al., 2017).

Last, many neurodiversity advocates (Silberman, 2015; Pantazakos, 2019), as well as scholars not explicitly affiliated with the neurodiversity movement (Martin, 2014; Lai and Szatmari, 2019), argue for shifting the focus of intervention from autistic individuals to their environment. According to the neurodiversity movement, neurodivergent disablement is indeed due to a mismatch between society and individual. Therefore, the idea here is that the physical and social environment should be partly transformed to accommodate neurodiversity. On the social level, such adjustments would include undoing prejudices, abolishing stigma, and gaining better understanding of autistics in public health, education, and society at large (Sonuga-Barke and Thapar, 2021). While perhaps not explicitly intending to, this approach can occasionally be seen to imply that the autistic individual’s environment should be the sole locus of intervention. The supporting argument would be that, since autism entails nothing inherently pathological, and since autistic suffering is due to social barriers, therefore the autistic person should not, qua autistic, become a clinical subject. In the next section, we argue why siding with neurodiversity proponents as regards the absence of inherent pathologies in autism does not, and should not, also necessitate supporting exclusively environmental interventions.

## 3. The need for neurodiversity-affirming clinical treatment

Neurodiversity-affirming autism clinical treatment, we argue in this section, should be prioritized as a matter of urgency. Our argument proceeds in four steps. First, clinical evidence clearly demonstrates that autistic people face a deep mental health crisis. Second, there is limited evidence to claim that this crisis can be overcome by targeting so called inherent, individual ‘dysfunctions’ within autistic people, as the medical model posits. Ongoing research rather indicates that adequate support structures are key for autistics mental health, much in line with neurodiversity proponents’ suggestions. Third, autistic people’s experiences within the mental health system in general are often alienating and counter-productive, reportedly because the system fails to observe autistic differences. Fourth, while perhaps historically and ethically understandable, antithesis to ABA in the form of a hands-off approach that advocates exclusively for environmental interventions leaves autistic people unsupported in areas crucial to their mental health and wellbeing. What is necessary, besides focusing on the physical and social environment, is rigorous, well-funded, neurodiversity-affirming clinical interventions, tailored to the needs of autistic people (Vanaken, 2022).

The magnitude of the mental health crisis among autistic people is hard to understate. A 2019 meta-analysis of 96 studies found that most psychiatric conditions are significantly more prevalent in the autistic than in the general population (Lai et al., 2019). Indicatively, pooled point prevalence estimates were at 20% (17–23, 95% confidence interval) for anxiety disorders; 11% (9–13) for depressive disorders; 9% (7–10) for obsessive-compulsive disorder; 5% (3–6) for bipolar disorders; and 4% (3–5) for schizophrenia spectrum disorders. For each of these conditions, point prevalence estimates were significantly higher than in the general population, with estimates for depressive and anxiety disorders, respectively, at double and triple rates (Lai et al., 2019). Another study following over 2.5 million individuals from 1987 to 2009 estimated that autistic people had a 2.56 higher odds to die in this timeframe than their non-autistic counterparts. This increased, premature mortality rate was found for nearly all causes of death. The mortality rate was even higher in the subgroup with a co-occurring intellectual disability, particularly regarding somatic causes of death. Alarmingly, 31% of premature autistic deaths was due to suicide compared to 4% in the general population. Suicide was the only cause of death more common among autistic people without versus with an intellectual disability (Hirvikoski et al., 2016).

While this type of evidence suggests a multi-faceted mental health crisis that befalls autistic people with disquietingly frequent dire consequences, considering additional evidence is essential in locating the root of the problem. According to the medical model, autism is an inherent pathology that differentially afflicts individuals, with the task of medicine being to address this pathology by reducing related symptom severity. If symptom severity was indeed solely to blame for the autistic mental health crisis, we would expect a positive correlation between the degree of autistic characteristics and mental plights. Existing evidence is at least inconclusive at this point, partly because cross-sectionally measuring mental health issues such as depression interferes with observations and self-reports of autistic characteristics (Hedley and Uljarević, 2018). In contrast to claims grounded in a medical model of disability, preliminary evidence points to a correlation between the wellbeing of autistic individuals and the perceived quality of support and the degree of social acceptance (Renty and Roeyers, 2006; Milton and Sims, 2016). Societal and parental acceptance also seem to be related to lower levels of stress and depression in autistic adults (Cage et al., 2018; Di Renzo et al., 2020). Qualitative research supporting the relatively new concept of ‘autistic burnout’ also corroborates this line of thought. Based on autistic people’s lived experiences, autistic burnout is conceptualized as the outcome of a sustained imbalance between the cumulative load of life stressors and the inability to obtain relief due to barriers to support (Raymaker et al., 2020). In line with these findings, neurodiversity scholars have often made the point that thriving and wellbeing are not necessarily at odds with being autistic, and that ‘a flourishing autistic life’ is not a contradiction in terms (Chapman and Carel, 2022). Overall, in agreement with social and interactional models of disability, we submit that there are firm and theoretically underpinned empirical reasons to believe that inherent autistic traits and related symptomatology are not the primary sources of the ongoing mental health crisis. Moreover, pursuing autistic-led development of support structures seems to be the foremost imperative for the autism clinic. Regrettably, this imperative is, as underlined in the previous section, at odds with the current allocation of autism funding. This is particularly problematic considering that autistic people’s encounters with the mental health system often prove unfruitful, and that neurodiversity scholars have frequently attributed such phenomena to the clinicians’ failing to grasp autistic subjectivity (Raymaker et al., 2017; Camm-Crosbie et al., 2019; Crane et al., 2019).

If support can make a substantial difference in autistic mental health, then it is worth looking at what kind of clinical support autistic people are currently getting. While we were unable to locate treatment patterns data for outside the U.S., the North American data speaks clearly to ABA being the dominant autism treatment. A 2018 U.S. study of over 43,000 children diagnosed with autism found that about 63% were receiving ABA (Xu et al., 2016). Another U.S. study of national insurance claims across two large online databases listing both adults and children found that behavioral therapy (including ABA) was by far the most common treatment (72–75%) for both adults and children (Shoaib et al., 2022). In addition, ABA for autism is the near-exclusive intervention that is being funded in North America (Gitimoghaddam et al., 2022). The therapeutic landscape might obviously look different in other Western countries, but it seems reasonable to say ABA is still at the core of the autism clinic, despite long-standing controversies.

As several others have discussed before, ABA is controversial regarding its efficacy -on its own terms- of diminishing autistic features and improving cognitive and language development (Sandbank et al., 2020), its biases and conflicts of interest in intervention research (Bottema-Beutel et al., 2020) and its poorly studied potential negative side effects (Dawson and Fletcher-Watson, 2021; Schuck et al., 2022). Yet, most importantly of course, are the conceptual critiques that ABA-inspired interventions aim to foster some form of typical functioning which is not a self-evidently desirable goal for autistic people (Dawson, 2004). Moreover, for our argument in particular there is good reason to believe that ABA-type of interventions may not succeed in addressing the autistic mental health crisis nor in bettering autistic lives in critical areas. Strikingly, there is a paucity in research documenting if and to what degree the many iterations of ABA may reduce autistic anxiety, depression, suicide risk, and address other facets of the autistic mental health crisis (Harvey et al., 2009). In addition, neurodiversity exponents widely argue that ABA negatively impacts autistic individuals. The most usual argument for this departs from the consideration that, since ABA explicitly targets autism ‘severity’ in terms of symptoms, and since these symptoms are in fact integral parts of the autistic self, ABA could therefore facilitate autistic camouflaging. In turn, camouflaging is known to be correlated with higher depression rates, reduced overall mental health, wellbeing, and increased suicidality (Cage et al., 2018; Cassidy et al., 2018; Hull et al., 2021). Generally, however, such claims are hard to assess because ABA’s harmful effects are scarcely measured, even regarding immediate impact (Dawson and Fletcher-Watson, 2021; Schuck et al., 2022).

The obvious objection to the above on behalf of ABA proponents would be that this type of intervention is not designed to address mental health problems, but instead to establish and enhance socially or developmentally important behaviors. This is a valid point, but it begs the question: is the teaching of these so-called important behaviors in turn conducive to either a mentally healthier life or a better life in other terms? As regards the mental health question, the answer, as we saw, is that we do not know. Given the depth of the mental health crisis that the autistic community faces, it seems problematic that the ‘gold standard’ for autism therapy leaves this question unaddressed. The same holds for the betterment of autistic lives in autistic terms. A recent scoping review of ABA interventions across seven online databases and systematic reviews found zero studies measuring the effect of ABA on subjective quality of life (Gitimoghaddam et al., 2022), which is again telling of a profound mismatch between dominant clinical interventions and the needs of autistic people themselves.

Let us pause and take stock. So far in this section, we have advanced three main arguments. First, the autism community is confronted with a far-reaching mental health crisis. Second, there is emerging evidence suggesting that this crisis can be ameliorated by the provision of proper support structures as defined by autistics themselves, rather than seeking to cure autism. Unfortunately, autistic experiences of the mental health system testify to such support structures being largely absent within it. Third, ABA, the most popular, at least in the U.S., intervention for autism is focused exactly on ‘reducing autism’. Further, ABA has a virtually non-existent evidential track record of ameliorating the autistic mental health crisis and bettering autistic quality of life. These considerations, we claim, prompt the conclusion that the funding, development, and provision of neurodiversity-affirming, autistic-tailored clinical treatment is urgent and of the essence if the pressing problem of the autistic mental health crisis is to be successfully tackled. In the next section, we explore what such treatment might look like in the flesh. In the remainder of this section, we will consider a possible counterargument to the above.

Previously, we pointed out a possible interpretation of the neurodiversity movement as advocating for exclusively environmental interventions. While well-intentioned and perhaps understandable in the light of the interventional torment to which autistic people have been subjected historically, a thus inspired course of action would be misguided. We hasten to note that by this we do not mean that environmental interventions should be discouraged. Such changes are indeed significantly helpful for mental health, as evidence suggests both regarding autism in particular (Milton and Sims, 2016) and marginalized groups in general (Mays and Cochran, 2011; Paradies et al., 2015). It is only making these interventions the sole focus of the autism clinic that we disagree with here. Realistically, in order to be effective, external interventions necessitate large-scale environmental adjustments and a remarkable shift in culture. Therefore, asking autistic people who are currently suffering to sit back and wait until such revolutionary restructuration takes effect to see a betterment in their mental health, even if their suffering is wholly due to social exclusion, is practically asking them to bear this weight unsupported. Almost needless to mention, it seems very unlikely that any individual’s mental hardships are wholly due to social exclusion. People ordinarily face at least some mental health challenges that are entirely unrelated to their exclusion from any social group, and there is no principled reason why these issues should eclipse in autism.

The case for the necessity of clinical treatment being more pronounced under conditions of social exclusion is implicitly or explicitly recognized regarding many other marginalized groups. To take two examples, it is well-known that Black and transgender people experience trauma and mental adversity due to racism and transphobia, respectively, (Mizock and Mueser, 2014; Comas-Díaz et al., 2019). This does not at any rate imply that clinical interventions are redundant to address such adversity or that the related clinic’s primary focus should be the undoing of racism and transphobia, respectively, – both these positions actually verge on the absurd. It is documented that Black and trans people benefit from and, one could argue, consequently deserve access to, additional treatment than white and cisgender people to deal with related trauma (Korell and Lorah, 2007; Comas-Díaz et al., 2019). Not only does marginalization imply psychological hardships in and of itself; it has also been argued that a minority identity will, due to mismatches with the majority, pose challenges even within a society not actively hostile toward one (Botha and Frost, 2018; Vanaken, 2022). In turn, quality identity-tailored and identity-affirming therapy will facilitate the processing and negotiation of one’s marginalized identity to mentally beneficial ends. If being autistic is a marginalized identity, as we saw influential neurodiversity scholars upholding over the previous sections, then it stands to reason that this should apply to autism as well.

## 4. The role of phenomenology

[R]ight from the start, from the time someone came up with the word ‘autism’, the condition has been judged from the outside, by its appearances, and not from the inside according to how it is experienced (Williams, 1996, 14).

Phenomenology is an especially polysemic philosophical tradition, minimally defined by describing and ordering encounters with the world from a first-person point of view. Phenomenology’s task is commonly viewed as one of locating the invariant structures of individual experience (Parnas and Zahavi, 2002), which will prove especially relevant for our discussion hereunder. The term ‘invariant structures’ denotes not the specific content of experience, but rather the form that a given subject’s experiences take. In the case of an autistic individual, this could be sensory hyper-sensitivity, or extraction of meaning in strictly literal terms (Pantazakos, 2019). Further, phenomenology is concerned with conscious phenomena’s personal significance, e.g., in a relational or sociocultural context (MacKinnon, 1993), as well as with their conditions of possibility, e.g., the bodily preconditions that must obtain for one to have a particular perceptual experience.

Phenomenologists have argued that emphasizing qualitative first-person analysis is of cardinal importance to medicine and the mental health professions (Parnas and Zahavi, 2002; Carel, 2011). The medical approach dominantly conceptualizes the body and the brain as physical machines, and medicine’s mission as repairing these machines in case of malfunction evidenced by neurological, biological, and/or behavioral means. However, for the medical patient or the client in therapy, the body and the brain are not experienced as physical objects but ‘from within’, in their qualitative immediacy (Toombs, 1987). Their condition is never for them a set of diffuse symptoms or localized organic functions, but a state holistically encompassing their lived experience. Moreover, the objective state of the body/brain has been argued to under-determine the corresponding subjective experience, sometimes substantially coming apart (Carel, 2011). Especially regarding the mental health professions, it is the case that brain states assume the importance they do only by virtue of their relationship with experiential mental episodes (Zahavi and Loidolt, 2021). Thus, the role of phenomenology within psychiatry and psychology is to clarify the experiential structures of conditions that fall under their rubric (Sass and Parnas, 2007). In turn, this knowledge can be employed both in diagnosis and in treatment, orienting clinical methods toward resonating in patient or client terms (Parnas and Zahavi, 2002; Carel, 2011). The significance of this endeavor has not been lost on the psychiatric canon, which is increasingly calling for phenomenologically informed approaches. The editorial of a recent volume of *The Lancet Psychiatry* (Boyce, 2021) reads:

Implicit in any phenomenological project in psychiatry or psychology is a shift in patient state from object of study to subject whose perceptions and experiences are heard, accepted, and valued. This approach could redress power imbalances, and generate collaborative projects in which all parties bring equally valued perspectives. Phenomenology cannot be the sole basis for mental health research. However, a greater number of dedicated investigations, and addition of a phenomenological dimension to larger projects, has the potential to advance – or at least unstick – multiple areas of mental health. There is no call to discard wholesale conceptual frameworks or the accumulated body of knowledge. But it is time to return to the things themselves.

Bearing in mind the discussion of the previous sections, the role we envisage for phenomenology is the clarification of the invariant structures of the autistic experience as part of an effort to adjust clinical treatment to the terms that such structures dictate. As we explain below, such a move is at the heart of developing much-needed neurodiversity-affirming, autistic tailored interventions. To begin, the primary phenomenological conclusion about autism is that it is characterized by markedly distinct first-person experiences. Related sources, affiliated with neurodiversity (Pantazakos, 2019) and beyond (Parnas et al., 2002; Turner-Brown et al., 2011), strongly argue that autism is a different mode of being in the world. Thus, autistic encounters with given stimuli, events, individuals, groups, institutions, and so forth cannot be unproblematically assumed to be like neurotypical ones. In turn, these experiences inform autistic people’s values, habits, ways of relating, and conception of the good life, which may diverge from neurotypical counterparts (Chapman and Carel, 2022).

Keeping this phenomenological divergence in mind, the first place to look for remedies for the mental ailments that autistic people face, e.g., anxiety and depression disorders, is clinical interventions that are the most efficacious against such ailments in the general population. Meta-analytic evidence demonstrates that, in general, principal interventions are pharmaceutical treatment and various forms of psychotherapy, such as cognitive behavioral, interpersonal, and psychodynamic psychotherapy (Leichsenring and Rabung, 2008; Cuijpers et al., 2011; Hoffman et al., 2012; Cipriani et al., 2018). Though we must point out a lack of research concerning how medication phenomenologically affects autistic individuals, further pursuing this direction is beyond the scope of this paper. Thus, we now turn our attention to phenomenology and psychotherapy for autism. In a 2013 special issue of *The Journal of Contemporary Psychotherapy* devoted to psychotherapy for autistic individuals, Koenig and Levine (2011, 31–36) highlight both the positive prospects of psychotherapy for autism, as well as the importance of phenomenology in realizing such prospects:

It is remarkable to be at the point where we are addressing what psychotherapy approaches might be best for working with individuals diagnosed with an autism spectrum disorder [sic] … improving quality of life and personal satisfaction from the standpoint of the affected individual has not been given the attention it deserves … Given the unique phenomenology of individuals with ASDs [sic], research-supported interventions will need modification and ongoing adjustments over time. … As psychotherapists, we may need to enlarge our conception of what “meaningful engagement” means in order to include and best serve individuals with social disabilities [sic], altering our previously established processes and rules for therapeutic engagement.

In the remainder of this paper, and in heartfelt agreement with this call, we develop a proposal of how phenomenological psychology can be mobilized to substantiate meaningful adaptation of contemporary psychotherapy to autistic terms. Here, we note that the intended audience of our suggestions is primarily non-autistic practitioners, a point to which we shall return below.

If one’s purpose is to tailor psychotherapy to autism, no place seems better to start from than neurodiversity-affirming clinical principles: engaging in participatory research and therapy; resisting normalization and respecting consent; and implementing environmental interventions. Since we are now dealing in individual psychotherapy terms, the last principle is not of immediate relevance. A common thread weaved through the first two is the call, best expressed by Sinclair (2012), to “not separate autism from the person.” The imperative is to not conceptualize autism as a pathological, removable thing that undermines the epistemic authority of autistic individuals and makes them blind to what is good for them. This has been expressed as the need to recognize a core autistic self^1^ within the individual (Sinclair, 2012; Pantazakos, 2019, 2023; Perrykkad and Hohwy, 2020), in terms of which therapeutic aims should resonate, and which should not be readily sacrificed at the altar of normalization as a set of problematic features.

At this level of abstraction, things seem relatively straightforward. Any psychotherapist who observes the neurodiversity paradigm will likely nod in agreement with the above. However, much as these principles are easy to agree on, they are also, in this form, more ethical guidelines rather than concrete clinical methodologies. Upon attempting to make the transition from the former to the latter, we claim, the picture gets significantly more complicated. The call to leave the autistic self as is, working instead on how the individual may achieve desirable outcomes in its terms, invites the question: how is one to distinguish between personality traits, behaviors, mental formations *et cetera* that should be ascribed to the autistic core self, and those that may be legitimately negotiated within therapy? Psychic phenomena certainly do not come pre-labeled as regards the substratum of the self that underpins them or lack thereof, and thus this question stands decisively in the way of neurodiversity-affirming psychotherapy.

At this point, it is worth looking into how the mental health professions tackle the question of psychopathology as regards the general population. The received way to define psychopathological cognitions, behaviors, and experiences is in terms of the ‘four Ds’: being deviant from the social norm; causing distress for the individual; causing dysfunction for the individual; and putting the individual and/or others in their environment in danger (Wilmshurst, 2015). The problem with these criteria is that, should one follow them in the case of autism, autistic cognitive and emotional styles are immediately classified as psychopathological. The obvious rebuttal from neurodiversity advocates would be that autistic cognitive and emotional tropes are not psychopathological but parts of the autistic self, and that psychotherapy should treat them as such. Deviation from the norm and from social expectations should be de-pathologized and left to be, and autism-related distress, dysfunction, and possible harm are not due to an inherent pathology, but mostly due to lack of support and society being ill-equipped to accommodate autistics. Consequently, should one be even minimally neurodiversity inclined, following the received methods of distinguishing between a harmless trait of the self and a problematic trait to be addressed in psychotherapy is unacceptable in the case of autistic clients. The dominant method of separating the self from the pathology slides us right back into the medical model, violating the neurodiversity paradigm’s most central claims. Still, all this leaves the question of identifying the autistic core self within the individual unanswered. This is a serious problem. As we saw, autistic clients report counter-productive encounters with mental health professionals, which are often ascribed to autistic subjectivity not being perceived and respected within the system. Failing to take in who the client is qua autistic will indubitably sabotage psychotherapy in particular as well, as therapeutic relationships and their success are inadvertently based on a foundation of understanding, which facilitates trust and a good rapport between therapist and client (Marziali and Alexander, 1991; Norcross, 2010). Hess (2009) has strongly claimed that a “sense-of-the-other” is at the core of all psychotherapy.

For these reasons, neurotypical practitioners could use all the assistance they can get while navigating who their autistic client is, and calibrating the changes they aspire toward while working alongside them. “Very well,” a neurodiversity proponent may now intervene, “here is your compass: just listen to whatever autistic people are telling you about who they are and what they want to change in their lives.” To be sure, feedback from the client is of paramount importance within every therapeutic intervention (Reese et al., 2009; Dyason et al., 2020). This is even more so in the case of autism, factoring in neurodiversity proponents’ convincing argumentation that autistic people have, to their detriment, been locked out of clinical design. Nonetheless, suggesting that a client’s declarations, especially regarding themselves, should be taken at face value and granted a status of ultimate authority within psychotherapy is overkill in the opposite direction. Interpreted literally, such advice is problematic and will end up only doing a disservice to autistic clients. Effectively all empirically credible psychotherapies contain a fundamental element of disbelief toward what the individual believes about themselves and/or of partly working against what comes naturally to them (Messer, 2002; Van Denburg and Kiesler, 2002). Notably, this regards all individuals, not just autistics or otherwise disabled. Arguably, if clients possessed perfect self-understanding, and if their habitual thinking and feeling patterns and behaviors were sufficing for their mental health and wellbeing, the need for psychotherapy would largely eclipse. Clients’ default modes, however, can and do fail them, and psychotherapy’s task is exactly to dismantle and unsettle these modes when this failure comes about. This, helping clients necessitates taking them seriously, not taking them literally and obediently. Therefore, a delicate balance must be found between respecting self-determination and challenging a client enough to overcome cognitive, emotional, and behavioral automations which may all but serve their mental wellbeing.

A hypothetical clinical example will serve both to make this point more tangible and to illustrate how phenomenology may help us overcome this impasse. Consider the following scenario: Jill, a college student, her neurotypicality or lack thereof unspecified, comes into a therapist’s office, the therapist being of any persuasion among the clinically dominant today. Jill’s presenting problem is protracted melancholia that seems to fit the profile of depressive disorder. Asked about her life, Jill mentions that she almost never socializes with her peers. She explains: “I naturally keep to myself. Besides, they all meet up in bars and clubs, and I cannot stand the lights and the loud music in there as anywhere.” Upon further inquiry, it is revealed that Jill has been raised in a single parent family, the primary caretaker being her father, who worked as a nightclub manager. Jill’s father used to regularly take Jill to work with him after school and leave her there unattended, resulting in her missing opportunities to socialize with children her own age and pursue extracurricular activities. Jill does not think that her upbringing has had a negative impact on her adult life. On the contrary, she believes that, despite difficult circumstances, she and her father managed to create a happy family. Following the initial exploratory sessions, the therapist decides to employ a double treatment method. First, to dialectically facilitate an exploration of Jill’s past and investigate a possible connection between Jill’s upbringing as a lonely child in a nightlife environment, and her current aversion to similar environments and hesitancy to socialize. Second, to prescribe a behavioral protocol with the purpose of progressively eradicating Jill’s aversive responses to nightlife contexts, thereby making her peers’ socializing domains feel more manageable to her. After a year in treatment, and despite the initial reading of her situation, Jill has come to establish a connection between her childhood, and her shyness and disdain of bars and clubs. Further, she has managed to successfully acclimate herself to such surroundings. She self-identifies as much happier than before the intervention, able to form meaningful connections and enjoy activities with peers. She no longer fits a depressive disorder diagnosis.

During this conjectured intervention, the therapist was operating on the assumption that sociability and peer group activities were things Jill longed for and could potentially enjoy. In other words, the therapist made the hypothesis that Jill’s refusal to socialize and her disinclination to nightlife were not part of her core self, but instead contingencies stemming from formative experiences. In turn, these experiences had cemented behavioral arcs leading to solitude, negatively impacting her mental health. Moreover, the therapist worked antithetically to Jill’s presenting sensory sensitivities, postulating either that they were reducible to other mental formations, or that what was to be gained from overcoming them was more important than the sensitivities themselves. Most importantly, by so doing, the therapist managed to steer the psychotherapeutic intervention so as to lessen suffering and bring more enjoyment in Jill’s life and in her own terms. There would be, we should think, virtually no disagreement with viewing this intervention as a successful one.

Imagine now an alternative outcome, one where the treatment course failed to provide results appreciated by Jill. It is *only* in this case, we maintain, that the treatment would have been a failure. If, by the end of the intervention, Jill was able to just withstand the music instead of enjoy it, mechanically talk to people but feel no satisfaction from such interactions, her depressive symptoms not having subsided, that would have plainly amounted to a waste of Jill’s and therapist’s time and resources. In this case, the therapist’s assumptions about the needs and joys of Jill’s core self would have been wrong, this evidenced via first-person, post-intervention investigation. This latter case of failure, we submit, captures the essence of what failed autism psychotherapy is. This is not with respect to Jill’s not actually wanting to socialize with her peers – autistic people often cherish socialization (Jaswal and Akhtar, 2018) – but regarding the therapist’s following supposedly universal assumptions about the human needs and joys, which are erroneously derived from neurotypical conceptions of what the good life is.

What we mean to convey with this story is that abiding by client self-narratives and behavioral defaults cannot tell good from bad therapy apart, neither for neurotypical people nor for anyone else. The same goes for normalization and reduction of the client’s epistemic authority, as both the good and the bad alternative outcomes above spring out of a common process resulting in Jill’s progressively adhering more to the social norm, while her self-descriptive statements were not admitted at face value – save for, very crucially, the stage of treatment evaluation. We rightly see none of these as problems in the good case scenario *only*, and this is precisely because Jill emerged out of therapy a person who was suffering less and enjoying more, experiencing an increased subjective quality of life. The value of a given psychotherapy regardless of neurotype, we contend, depends foremostly on the fulfillment of this condition. To be sure, there are hard limits to therapeutic interventions. Change cannot be forced but only pointed to and facilitated, if not for reasons effectiveness, then surely for reasons of respecting consent and human rights within the clinic. Beyond this minimum, however, the end justifies the means, so long as the end is defined on client terms.

To transpose our conclusions to the case of autism, we claim this: as neurodiversity exponents would suggest, testimony from the autistic client can indeed tell us which cognitive, emotional, and behavioral tropes belong to their core, autistic self, and which are inviting change via psychotherapeutic intervention. However, admitting such feedback uncritically and based on the clients’ automatic modus operandi will only hamper psychotherapeutic effectiveness. The kind of feedback that will serve to pinpoint the autistic self, and by extension guide autistic psychotherapy productively, is phenomenological feedback on the effects of specific psychotherapeutic directions. In this view, a given change is aligned with the autistic self if it is meaningful once it is successfully brought about. Conversely, a change is incompatible with the autistic self if it is not meaningful post-integration. ‘Meaningful’ is here cashed out in terms of facilitating a happier and less distressed life, this judged from a phenomenological point of view. We may now express neurodiversity proponents’ criticisms of ABA as claiming that such meaningfulness-oriented phenomenological feedback is not actively sought for, or even altogether ignored within the clinic. Therefore, we contend, the neurodiversity movement’s clinical position should not be that autistic people can wholly and successfully direct their own therapy. Instead, the more subtle demand should be that clinicians immerse themselves into autistic phenomenology and pay due credit to autistic first-person experiences, especially regarding the meaningfulness of procured results. In turn, phenomenology, as the foremost method for describing human experience and investigating meaning structures in the first person, is also the primary tool for articulating the effect of, methodologically informing, evaluating, and choosing between, autism clinical therapies.

Phenomenology scholarship provides several toolkits for recording an individual’s experience in a clinical context and/or a context of disablement or psychopathology (Parnas et al., 2005; Carel, 2012; Schmidt, 2018). Of special relevance to the discussion conducted herein is interpretative phenomenological analysis (IPA, Smith et al., 2009). IPA is a qualitative research method that involves a comprehensive examination of personal lived experience. It mobilizes instruments such as interviews, diaries, and focus groups to facilitate an individual’s detailed first-person investigation of their own experiences. In the most usual case of interviews, the participant is asked questions pertinent to the research topic and encouraged to provide as full an account of their experience as possible, while their answers are, ideally, transcribed verbatim. Following, the researchers analyze the recorded data for key themes, bracketing, to the degree achievable, prior hypotheses and preconceptions about what will emerge. The themes’ purpose is to identify what matters to the participants in given experiential contexts, as well as to pin down the specific meaning of points of importance. During this process, ‘superordinate’ themes may ensue, which signify overarching patterns of meaning in the interviewee’s life. Last, a report of the resulting themes is produced, usually regarding several individuals. Frequently, the final identification, interpretation, and discussion of themes incorporates feedback from, or even necessitates the final approval of, the interviewee(s).

A key feature of IPA that distinguishes it from other qualitative methods is its ‘double hermeneutic’ method. Within the double hermeneutic, researcher and subject engage in a process of intersubjective sense-making, whereby the subject is making sense of their own experience, while the researcher is making sense of the subject’s account (Montague et al., 2020). Thus, IPA views participating subjects as experts on their own experience, placing itself ideally to address the double empathy problem between neurotypical researchers and autistic subjects (Milton, 2012), as well as the marginalization of autistic voices within autism research and clinic (Howard et al., 2019). Additionally, IPA has already been partly adapted for autistic participants regarding ascertainment of consent and minimizing distress (Huws and Jones, 2015); recognition of the need for alternative to the interview platforms for some autistic individuals (Humphrey and Lewis, 2008); and accommodation of non-verbal autistic communication via the employment of, e.g., drawings and photo elicitation (King et al., 2017). Further, IPA is argued to be especially valuable when examining topics that are ambiguous, elusive, and emotionally laden (Smith and Osborn, 2008). Making sense of how a treatment has impacted an individual is precisely such a complex issue.

Our proposal here is to employ IPA, and other methods of phenomenological inquiry possibly fitting the purpose, for interrogating the impact of psychotherapeutic interventions on autistic individuals. The hypothesis is that this will make for treatments multiply more beneficial to autistic people than the currently available, thus addressing at least part of the ongoing autistic mental health crisis. The proposal on offer constitutes both a research program at large and a methodology for individualized implementation within specific therapist-client interactions. First, phenomenological research should investigate which particular environments, therapeutic assumptions, methods of interaction, goal-setting strategies *et cetera* result in optimal first-person results for autistic people. To the extent that the autistic self is non-homogeneous, we should reasonably expect to find that different things work for different people. At the same time, to the extent that autism constitutes a distinct, despite heterogeneous, mode of being, one should be able to draw the contours of effective therapeutic stratagems from structural experiential commonalities across the autistic population. Second, knowledge resulting from this research should be brought to bear on individual therapeutic collaborations. The therapist should be aware that phenomenological evidence about what generally works for autistic people is *indicative*. Just as one cannot expect any prototype of the neurotypical self to fit all neurotypical clients, so one should expect their phenomenologically-informed-for-autism stratagems to require tailoring to the individual client to prove successful. This may be achieved via intermittent phenomenological feedback sessions nested within the therapeutic trajectory (see next section).

This is, in essence, what we envision as phenomenology and neurodiversity foundations for autism-related psychotherapy. The benefit of this approach is that it prioritizes autistic terms in keeping with neurodiversity, while at the same time proposing a concrete methodology and refusing to make compromises that would be antithetical to fundamentals of therapeutic potency, i.e., challenging the client. Provided that such challenging proves to have a beneficial effect, the above implies nothing prohibitive for running against the default mode of the client.

## 5. Phenomenology and neurodiversity in current clinical methods

The proposal advanced in the previous section will naturally require substantial localization and further specification to be integrated within existing clinical schemes. In this section, we develop, to the limited extent possible herein, recommendations on how two of the most popular and efficacious kinds of psychotherapy – cognitive behavioral (CBT) and interpersonal (IPT) – may incorporate the framework outlined above. To provide concrete examples of how these therapies can adjust to autistic clients’ needs, we refer to available phenomenological evidence that relays autistic experience in general. As per the previous section’s proposal, phenomenological inquiries regarding the autistic experience of the specific therapies in question is encouraged.

CBT and IPT define several intervention stages (Stuart and Robertson, 2003; Beck, 2011). Both approaches include initial exploratory sessions, where the therapist assesses the case, familiarizing themselves with the client’s presenting problems, symptoms, and history. In CBT, the client’s problems are conceptualized on a cognitive, emotional, and behavioral level. The therapist works with the client to set specific and measurable goals, exploiting cognitive (e.g., cognitive restructuration) and behavioral (e.g., behavioral experiments) techniques to achieve them. The therapist provides ongoing feedback on the client’s progress and, following treatment, collaboratively sets up prevention and maintenance mechanisms for ongoing self-monitoring and care. In IPT, client problems are articulated within a relationship framework and understood in terms of interpersonal conflicts. As in CBT, treatment aims to achieve tangible goals. Relevant techniques include the development of new skills and strategies for improving relationships, such as clarifying client emotions and finding healthier ways to express them, improving communication skills, setting boundaries, and solving problems. Following treatment, therapist and client work together to ensure that the client will be able to continue to utilize skills and resources to maintain their progress post-treatment. Both CBT and IPT are kinds of psychotherapy that rely relatively strongly on verbal communication and cognitive and emotional self-reflection. Therefore, this might potentially conflict with autism’s co-occurrence with language impairments, intellectual disability and alexithymia. Below, we return to these limitations, yet for now, we want to stress that CBT and IPT merely function as examples to concretize our proposals for a phenomenologically-informed, neurodiversity-affirmative kind of psychotherapies which stretches beyond these examples of talk therapy.

CBT has begun to attract the attention of autism clinicians. There is meta-analytic evidence to suggest that CBT is effective for treatment of autistic anxiety (Lang et al., 2010) and, though evidence is more limited, the same may be true for depression (Pezzimenti et al., 2019). CBT therapists report routinely making adaptations to their practice when working with autistic clients, but also admit limited confidence regarding their ability to bring such adaptations about successfully. The literature includes both calls to systematically tailor CBT to the special treatment needs of autistic people, as well as limited concrete attempts in this direction (e.g., Sze and Wood, 2008). In contrast, research concerning IPT in connection with autism is virtually non-existent, as are attempts to adapt IPT to autistic individuals. This is remarkable because the subject matter of IPT – the improvement of mental health through the betterment of interpersonal relationships – is a natural fit for a defining characteristic of autism, namely problems in connecting with others (American Psychiatric Association, 2013).

Phenomenology, we claim, is an indispensable tool for furthering and systematizing the CBT-autism connection, and for establishing one between IPT and autism. To explicate how, we underline frequent characteristics of the autistic experience that likely pose a challenge for the implementation of CBT and IPT for autistic people. Such characteristics include: sensory overwhelming within the clinical context; overbearing social interaction with clinicians; reduced ability to manage abstract concepts (e.g., thinking errors and cognitive distortions); facing demands for unachievable cognitive flexibility and perspective-taking (Minshew et al., 2002; Koenig and Levine, 2011; Leung and Zakzanis, 2014; Cooper et al., 2018); difficulty with interpreting social cues, facial expressions, and nonverbal communication; building and maintaining relationships; and dealing with expectations around social interaction (Travis and Sigman, 1998; Koenig and Levine, 2011; Badder and Fuchs, 2021).

Phenomenological input in the style of IPA will help navigate related challenges across all stages of treatment. Starting from the clinical setup, both CBT and IPT alike should observe already available evidence that relates to particular sensory and cognitive tropes of autism (Narzisi and Muccio, 2021), ascertaining that the therapeutic environment, the session duration, and the communication style of the therapist are adjusted according to the needs of the client (e.g., employing low lighting, absence of loud sounds, literal language). The conceptualization and problem identification phase of treatment should follow autistic phenomenology as well. For example, absence of socialization to a degree characteristic of neurotypical peers should likely not be conceptualized as a problem in and of itself. First-person investigations of autism strongly support that autistic people often experience a burnout state as a result of being overtaxed by demands that are out of sync with their own social needs (Arnold et al., 2013). To take another example, should a client claim that bright open plan offices are too sensorily overwhelming for them, the therapist should in all probability not construe this as an individual problem, as autistic phenomenology testifies to such environments often being extremely unpleasant to autistic people even after acclimatization (Booth, 2016).

Correspondingly, the implementation stage of both treatments, and the strategies for achieving set goals, should also be guided by autistic phenomenology. In the case of CBT, phenomenological inquiry sessions, nested in-between regular sessions should ascertain that the cognitive and behavioral stratagems initially deployed do not turn out to be senseless, impossible to follow, or too distressing for the client. As noted previously, some autistic people may find certain exposure protocols insufferable and/or exhibit characteristics (e.g., limited cognitive flexibility) that render given assignments (e.g., cognitive restructuration) meaningless. In both these cases, the therapeutic stratagems should be abandoned, for reasons of effectiveness and avoiding harm, respectively. Concerning the bright open plan offices example above, avoiding addressing employment problems by attempting to acclimatize the client to the work environment, and pursuing other alternatives instead, is advised. More generally, the therapist should keep in mind that phenomenological autistic testimony has established that behavioral ‘adjustment’ often amounts to non-meaningful results, masking, and correlated adverse effects.

In IPT, insisting on repairing a relationship with a neurotypical partner or friend by spending more quality time with them is not advisable beyond a temporal limit that distresses the client, as pushing against a client’s socialization comfort zone may exacerbate the effects of autistic burnout. Forging closer connections through eye contact and joint attention should likely not be pursued, seeing as there is phenomenological evidence substantiating that such aversions are often part of the autistic self, i.e., that overcoming them does not make for connections that are experienced as more meaningful by the autistic client (Trevisan et al., 2017). Should an autistic client exhibit a behavior that might seem antisocial to most others but be essential to the client for purposes of self-regulation, then pushing against that behavior would also be inadvisable. Phenomenological accounts suggest that self-regulatory behaviors that may seem ‘weird’ to others can be essential to autistic self-regulation (Bascom, 2011; Kapp et al., 2019). Instead of attempting to change such behaviors, it may be recommended to the client that they inform their peers about them and their meaning pre-emptively, asking peers to not interpret, e.g., stimming as a sign of alarm or of personal rejection. A departure from traditional concepts of friendship may be necessary, as there is evidence to support that, for autistic children, meaningful friendship tends to revolve around activity rather than emotion sharing (Bauminger and Kasari, 2000).

Expectedly, feedback is the primary locus of phenomenological intervention in both CBT and IPT. Feedback should not be given just from the side of the therapist and regard only measurable progress on pre-defined goals. It should also be provided from the side of the client and convey how experience of the world, their own selves, their relation to others, and their environment has changed in the course of therapy. The efficacy of any CBT and IPT program should not be rated based on observable results alone, such as reduction of autistic symptoms and increase in number of friends and frequency of interactions with peers. These should be considered vacuous if failing to correspond to improvements in mental health and subjective wellbeing. Changes should be assessed on the basis of their meaningfulness from the client’s point of view. Thus, IPA, with its emphasis on clarifying meaning structures in subjective terms, is particularly well-suited to the purpose.

Crucially, and to highlight the previous section’s point, these client-steered modifications should not be materialized with an attitude of blindly following all client self-declarations. Indeed, CBT and IPT therapists should do well to explore avenues that may run contrary to the client’s defaults, while, of course, respecting their consent. Adjusting to the client in the *specific* respects mentioned above is advisable because phenomenological research demonstrates that resistance from the side of the client is in all likelihood due to non-adjustable core self traits. Equally importantly, therapists should not *a priori* assume that the above is applicable to all clients; treatment should be individualized by acquiring personal phenomenological testimony. Upon, however, encountering a typical autistic phenomenological feature, the therapist should ascribe increased probability to its not being a problem or symptom to be altered, but a core feature of the self that the treatment needs to take as a point of departure.

Here, a complication may arise, important and under-emphasized in neurodiversity treatises of the autism clinic, namely that some of an autistic person’s priorities and desires may be dystonic to their neurotype (Chapman and Botha, 2022). For example, an autistic person may long for group interactions with neurotypical peers, but at the same time find the environments where this socializing happens overbearing. It may also be unrealistic to expect that this group of peers adjust their socializing environments to the client’s needs. In the case of such contradictions, therapist and client may decide to work against a trait of the client’s self to achieve especially cherished outcomes. Very importantly, the result of this strategy and its meaning should also be phenomenologically evaluated – was, in the client’s own understanding, achieving socialization endurance worth the trouble of sensory distress? Running against the self should be done only when conferring benefits that outweigh distress, this judged from a phenomenological perspective.

Before drawing this section to a close, a few general notes concerning our proposal. First, we do not remotely pretend to have covered all ways in which CBT and IPT may be improved upon phenomenologically and for autism, or to have provided sufficient details to make our proposal ready for clinical application. Rather, our purpose in the previous section was to provide indicative ways of grounding the research program on offer. Comprehensive phenomenological research, and further specifying its application within the clinic, are thus the subject of future research into the issue. Second, we have here covered only a few types of therapy applicable to autism, while several other candidates not infrequently used, such as mindfulness-based approaches and social skills interventions (Kang et al., 2022), were left unexplored, just as therapies that are less verbal in nature. We have chosen CBT and IPT to make a first pass at for phenomenological adaptation because the issues they address seem to us particularly relevant to autism-associated mental hardships, without meaning to undervalue suchlike adaptation of other forms of treatment. Third, and potentially most importantly, an increased presence of autistic therapists within therapeutic communities will likely be very helpful in the phenomenological attunement of treatment to autistic people. Recall that the question at the root of the present discussion regarded telling the core autistic self apart from contingent elements. Phenomenological feedback was introduced as the primary way of addressing this problem. An alternative, or better yet complementary, approach would be to go in from the ‘front end’ of the problem, matching autistic clients with therapists that are more naturally posed, due to personal experience, to be phenomenologically acquainted with the autistic self.

Last, two limitations. First, the above proposal turns foremostly on autistic people’s self-reporting of their emotions: whether, to what degree, and how their self and their interactions with the world feel different post-treatment. It is well-evidenced, however, that autistic people often exhibit alexithymia, with a 2020 meta-analysis estimating the related prevalence rate at about 50% (Kinnaird et al., 2019). Alexithymia is a little-understood psychological phenomenon that involves difficulties in identifying emotions experienced by oneself and others (Larsen et al., 2003), and is associated with physical and mental health impairment and poor outcomes in at least some forms of psychotherapy (Cameron et al., 2014). Thus, it may be argued that alexithymia is worrisome for both autism psychotherapy in general, and its phenomenological evaluation in particular. This point is valid but notice three things. First, there is evidence to suggest that psychotherapy is itself beneficial to clients exhibiting alexithymia (Cameron et al., 2014). Second, this problem is not at all specific to autism, as about 25% of people who seek psychotherapy are considered to be alexithymic (Grade et al., 2008). Third, it has been suggested that poor treatment outcomes in alexithymic clients can be partially explained by therapists negative reactions to the clients limited abilities to read and express their emotional states, leading to a poor therapeutic reliance (da Silva et al., 2018). We hypothesize that taking a more phenomenological approach to psychotherapy can actually help overcome this barrier.

Second, it is well-known that about one third of autistic individuals are also diagnosed as being intellectually disabled (Center for Disease Control and Prevention, 2023). It is also estimated that about 25–50% of autistic children do not develop functional verbal communication (Patten et al., 2013). As regards the former, psychotherapeutic efficacy for individuals diagnosed with intellectual disability is controversial, with theoretical frameworks and empirical data pulling in opposite directions (Sturmey, 2005; Taylor, 2005). As regards the latter, there exist forms of psychotherapy purportedly fitting the needs of nonverbal people, such as art and play therapy, though their outcomes are extremely understudied. Regardless of whether traditional forms of psychotherapy can be beneficially adjusted in either of the two cases or whether radically different forms of therapy should be followed, we believe that the essence of our phenomenological proposal carries over. The evaluation of any therapeutic program should be result-centered and predominantly carried out in the client’s terms, whoever this client may be. Intellectual disability and nonverbal communication may stand in the way of specific forms of psychotherapy like psychodynamic psychotherapy and IPT, but these factors do not obviously challenge the proposal to phenomenologically tailor therapy to autistic needs. Fortunately, as we saw earlier, IPA has been successfully adapted to record phenomenological responses from a variety of people, including those who do not use verbal language to communicate.

## 6. Conclusion

We began this article by reviewing clinical evidence demonstrating that autistic people face a mental health crisis. Though autistic symptomatology does not appear to be the culprit for this crisis, research priorities and the biggest share of autism funding are oriented toward understanding the biological foundations of autism. This, as neurodiversity proponents point out, leaves a gap in the research and development of services tailored to the needs and priorities of autistic people themselves. Moreover, ΑΒΑ, the treatment supposedly best fitting autism, is entirely unevidenced to aid with the mental health ailments autistic people face, and autistic people’s experiences with the mental health system are generally discouraging and counterproductive. This picture, we argued, implies that the development of autistic-tailored mental health clinical treatments is of the essence. We then put forward a proposal elaborating how result-centered phenomenological inquiries into existing clinical programs can be utilized to develop neurodiversity-affirming therapies. The hypothesis is that such treatments can fill the autistic mental health support lacuna, thus responding to the related mental health crisis. Further details implementing this project at the level of individual clinical schemes are left to future research, as is empirical confirmation of all hypotheses advanced herein.

## Data availability statement

The original contributions presented in the study are included in the article/supplementary material, further inquiries can be directed to the corresponding authors.

## Author contributions

TP conceptualized and researched the main arguments, claims presented in this manuscript, drafted the initial version of the manuscript, held overall oversight and responsibility for the manuscript while collecting and implementing feedback from colleagues and the co-author. G-JV had general oversight of reviewing the clinical data, commented on the initial version of the manuscript, suggested several conceptual changes and additional literature to included, and engaged in co-authoring the final version of the manuscript. All authors contributed to the article and approved the submitted version.

## Funding

This work has received funding from the European Research Council (ERC) under the European Union’s Horizon 2020 research and innovation programme (Neuroepigenethics project), acquired by Kristien Hens, grant agreement No 804881.

## Conflict of interest

The authors declare that the research was conducted in the absence of any commercial or financial relationships that could be construed as a potential conflict of interest.

## Publisher’s note

All claims expressed in this article are solely those of the authors and do not necessarily represent those of their affiliated organizations, or those of the publisher, the editors and the reviewers. Any product that may be evaluated in this article, or claim that may be made by its manufacturer, is not guaranteed or endorsed by the publisher.

## Footnotes

^1^There are readily available philosophical complications that pertain to the concept of the self, such as how the self is defined, whether humans have a single self or many and contextual, if and how the self evolves in time *et cetera*. Obviously, these questions cannot be addressed herein, and thus we take the concept of the autistic core self to signify a set of core traits of the autistic individual.
